# FRAMM: Fair ranking with missing modalities for clinical trial site selection

**DOI:** 10.1016/j.patter.2024.100944

**Published:** 2024-03-01

**Authors:** Brandon Theodorou, Lucas Glass, Cao Xiao, Jimeng Sun

**Affiliations:** 1 University of Illinois at Urbana-Champaign, Urbana, IL, USA; 2 IQVIA, Durham, NC, USA; 3 GE Healthcare, Seattle, WA, USA

**Keywords:** trial site selection, fairness in healthcare, deep learning, machine learning for healthcare, missing data, reinforcement learning, learning to ranking, fairness in machine learning

## Abstract

The underrepresentation of gender, racial, and ethnic minorities in clinical trials is a problem undermining the efficacy of treatments on minorities and preventing precise estimates of the effects within these subgroups. We propose FRAMM, a deep reinforcement learning framework for fair trial site selection to help address this problem. We focus on two real-world challenges: the data modalities used to guide selection are often incomplete for many potential trial sites, and the site selection needs to simultaneously optimize for both enrollment and diversity. To address the missing data challenge, FRAMM has a modality encoder with a masked cross-attention mechanism for bypassing missing data. To make efficient trade-offs, FRAMM uses deep reinforcement learning with a reward function designed to simultaneously optimize for both enrollment and fairness. We evaluate FRAMM using real-world historical clinical trials and show that it outperforms the leading baseline in enrollment-only settings while also greatly improving diversity.

## Highlights

• The underrepresentation of minorities in clinical trials is a big problem
• We propose FRAMM to guide fair trial site selection to address this problem
• FRAMM uses reinforcement learning to optimize for both diversity and enrollment
• On historical trials, FRAMM boosts minority enrollment by up to 20% for some groups

## The bigger picture

The issue of underrepresentation of gender, racial, and ethnic minorities in clinical trials is a long-standing and severe problem. This gap in enrollment has historically led to treatments that are less effective for these groups, highlighting a significant challenge in achieving equitable healthcare outcomes. By proposing FRAMM, a deep reinforcement learning framework for fair trial site selection, we take a significant step toward more inclusive and effective medical research. FRAMM’s ability to simultaneously optimize for both enrollment and diversity helps to ensure that clinical trials more accurately reflect the diverse populations that they aim to serve, without sacrificing the overall enrollment necessary to make them work. While this paper serves mainly as a case study and proof of concept, the ambitions are to transform both the ideology and methodology of trial enrollment and research to make it more equitable and fair at every step of the way.

## Teaser

FRAMM, a cutting-edge machine learning framework, addresses the critical issue of minority underrepresentation in clinical trials by innovating fair trial site selection. Using deep reinforcement learning, FRAMM adeptly navigates incomplete data and simultaneously optimizes enrollment and diversity. Tested on real, historical trials, it not only surpasses existing methods in enrollment efficiency but also significantly enhances participant diversity, paving the way for more inclusive and effective healthcare research.

## Introduction

Clinical trials are the currently widely accepted process for evaluating the efficacy and safety of proposed new treatments for diseases. Enrolling sufficient patients from all gender, racial, and ethnic groups is essential for ensuring the treatment’s efficacy on all groups. Despite many efforts to address the disparities,^1^^,^^2^ the underrepresentation of minorities in clinical trials remains a problem.^3^^,^^4^ This consequently undermines the fairness for minorities in obtaining effective treatments. For example, reports show that African Americans make up 13.4% of the US population, but only 5% of trial participants. Hispanics represent 18.1% of the US population, but less than 1% of trial participants.^5^ These gaps can then have drastic and problematic downstream effects due to well-documented racial and ethnic differences in response to medicine.^6^ Without representation of these groups, we are unable to obtain precise estimates of treatment effects within different subgroups and thus unable to guide both the development and use of medicine with these groups in mind.

To address the enrollment disparity, existing efforts have included government policy,^7^ softening eligibility criteria to make trials more accessible,^8^ and a community engagement-based approach.^9^ Recently, deep learning has been introduced to site selection. For example, Doctor2Vec^10^ proposed to select sites based on predicted patient enrollment. This development brings promise to scalable site selection, but it does not yet have any consideration for diversity.

This is especially important as historical realities make the problem of site selection necessarily a trade-off between enrollment and diversity. While there certainly exist diverse and high-enrolling sites, the inequities in past enrollment and data mean that most previous sites that would typically be selected again are predominantly White. So, we must by definition go away from these predictably high-enrolling sites to introduce greater diversity. Furthermore, we must do so within the constraints of a fixed or at least limited number of sites due to the unpredictability of enrollment and the huge cost of approaching new sites to enroll further patients.^11^ Such realities make approaches such as simply adding additional, diverse sites or imposing enrollment caps to limit majority group patients undesirable as they are costly and limit overall enrollment, respectively. Thus, there are still the following challenges to be solved.

(1) **Missing data modalities across sites.** Different trial sites can have different modalities of features that can be predictive of patient enrollment when seeking to pick sites for future trials. Some of these feature modalities such as claims or clinician specialty data can also be missing at different sites. Trial sites with a greater minority population are more likely to have missing data due to insufficient data collection and reporting, so failure to handle this problem only exacerbates the underlying unfairness.
(2) **The enrollment-diversity trade-off.** While selecting sites only to maximize enrollment can be treated as a prediction task, the addition of diversity adds a unique challenge to the problem. We can not simply constrain fairness by setting minimum percentage thresholds for each group because they would effectively set enrollments caps due to the low minority population selected by enrollment-only models, and the administrative constraints limiting the overall number of sites we can choose. So, the problem is necessarily a trade-off between enrollment and fairness, and we thus need to optimize simultaneously for both objectives.

**Figure 1:**
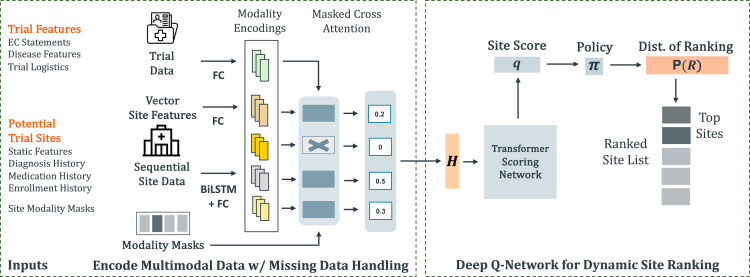
A visualization of the FRAMM framework FRAMM uses multi-modal site features and the trial representation to generate scores for, rank, and select a subset of prospective trial sites. The pipeline used to do so consists of modality encoders, a missing data handling mechanism, a scoring network, and a reinforcement learning-based ranking approach.

To address these challenges, we propose a deep reinforcement learning framework named FRAMM, shown in Figure 1, which is enabled by the following technical contributions.

(1) **Modality encoder for missing data handling.**FRAMM handles missing data by first mapping all the different feature modalities in their diverse data formats into a shared, uniformly formatted representation space. It then combines the present modality representations into a single site representation via a masked cross-attention mechanism. This missing data module uses the trial as a query to build a site representation without needing complete site features.
(2) **Deep reinforcement learning for efficient trade-offs.**FRAMM is equipped with a deep reinforcement learning setup with a specifically built reward function that simultaneously optimizes for both enrollment and fairness metrics. It also has a deep Q-Value network that approximates the contribution of each individual site to the corresponding reward given their site features.

We evaluate FRAMM using 4,392 real-world clinical trials ranging from 2016 to 2021 from a large clinical trial company. We show that FRAMM outperforms the leading baseline in enrollment-only settings while also achieving large gains in diversity. Specifically, it is able to produce a 9% improvement in diversity with similar enrollment levels over the leading baselines. That improved diversity is further manifested in achieving up to a 14% increase in Hispanic enrollment, 27% increase in Black enrollment, and 60% increase in Asian enrollment compared with selecting sites with an enrollment-only model.

### Background and related work

#### Machine learning for clinical trials

There have been a number of recent applications that look at using machine learning to optimize clinical trial operations. These include matching patients to trials that they are eligible for,^12^^,^^13^^,^^14^ searching for similar trials,^15^ and predicting trial outcomes.^16^ There have even been some works seeking to predict site enrollments to help select trial sites.^10^^,^^17^ However, none of the existing works were designed to optimize enrollment diversity.

#### Fairness in machine learning

Nevertheless, fairness more generally has been a well-explored topic in the wider machine learning domain. Many applications within the area (see surveys^18^^,^^19^^,^^20^) seek to limit or eliminate biases within the model inputs via preprocessing or model outputs via post-processing. However, our application belongs to the third class of fairness-based models, in-processing, which reduces biases and improves fairness during training via approaches such as adversarial learning,^21^ bandits,^22^ regularization,^23^ and re-weighting.^24^ Specifically, we seek to utilize a constraint optimization approach to in-processing as in Zemel et al.,^25^ Nabi et al.,^26^ Celis et al.,^27^ and Narasimhan et al.^28^by tapping directly into the learning framework and adding a fairness component to our loss function. However, our framework FRAMM is unique even in comparison to these similar approaches as we avoid directly learning within some fairness constraint but explicitly optimize and maximize fairness metrics.

#### Ranking methods

The standard ranking problem is a well-study topic.^29^^,^^30^^,^^31^ Despite a good amount of literature dealing with fair ranking, most approaches constrain fairness to an acceptable level,^32^^,^^33^^,^^34^ ensure certain protected features do not have an impact on any model outputs,^35^^,^^36^ or add regularization to the loss function^36^^,^^37^ rather than seeking to maximize fairness metrics as we do.

In particular, our approach uses reinforcement learning (RL) to rank with an abstract reward function, which can be made to incorporate fairness metrics. This is an especially exciting subdomain of the learning to rank problem with numerous promising approaches^38^^,^^39^. Liu et al.^40^ use a fairness-dependent loss function in a different setting involving recommendations over time. Feng et al.^41^ introduce a multi-agent setup designed to optimize multiple objectives. Clark and Manning^42^ and Wei et al.^43^ present new policy gradient algorithms used to train their ranking models. However, while RL-based approaches are flexible enough to support our goal, our problem setting of explicitly optimizing for fairness with missing data is still a unique foray into the field.

#### Missing data handling

Classical missing data handling mainly relies on data imputation. Most existing methods typically reconstruct embeddings of missing modalities based on the embeddings of present ones as in Ma et al.,^44^ Tran et al.,^45^ and Lau et al.^46^This requires some complete data points and pre-training of the imputation model. Other approaches include modality dropout during training,^47^ or learning multiple conditional distributions for different combinations of present modalities.^48^ However, alongside the growth in the popularity of attention mechanisms within architectures such as Transformers, there has also come an exploration of how attention, with built-in support for masking certain inputs can be used to handle missing data.^49^^,^^50^^,^^51^ Our framework leverages a similar approach via a cross-attention mechanism that allows us to entirely bypass data imputation and the need for complete data in model training.

## Results

### Problem formulation

#### Clinical trial site features

For each clinical trial, the trial is represented by a vector $t\in R^{n_{t}}$ containing features for things such as disease information, trial logistics, and eligibility information. Its sites may then contain any number of features in a variety of different modalities depending on the available data. In our dataset, our sites correspond to individual clinicians or investigators, and they are represented by the following data modalities:

(1) **Static information**$s_{i}\in R^{n_{s}}$ is a vector representing a site’s primary clinician’s gender, profession type, primary specialty, patient demographic distributions, and the geo coordinates of the site.
(2) **Diagnosis history**$D_{i}\in R^{n_{c}\times n_{d}}$ is a sequence of $n_{c}$ one-hot vectors representing the ICD-10 codes (out of $n_{d}$ options) of the diagnoses of the most recent patients for a given site.
(3) **Medication history**$P_{i}\in R^{n_{c}\times n_{p}}$ is a sequence of $n_{c}$ one-hot vectors representing for the Uniform System of Classification ontology level 2^52^ codes (out of $n_{p}$ options) of the most recent prescriptions at a given site.
(4) **Enrollment history**$E_{i}\in R^{n_{h}\times \left(n_{t}^{\prime }\right)}$ is a sequence of $n_{h}$ trial representations (omitting the inclusion/exclusion criteria here due to dimensionality/memory concerns) and enrollment numbers for the most recent trials of a site.

In addition, trial sites also have an input feature mask $m_{i}\in R^{4}$ represented as a 4-dimensional binary vector signifying whether each feature modality is present or missing. Finally, trial sites are labeled using their enrollment value for the trial $e_{i}\in R$ and their 6-dimensional vector $r_{i}\in R^{6}$ representing their racial distribution. For example, if site 4 enrolled 95 participants and has a racial makeup of 47% White, 23% Hispanic, 15% Black, 10% Asian, 4% Mixed, and 1% Others, we have $e_{4}=95$ and $r_{4}=\left[47,23,15,10,4,1\right]$.

So, each trial t is scored against *M* trial sites, which are each represented as $S_{i}=\left(\left(s_{i},D_{i},P_{i},E_{i}\right),\left(m_{i}\right),\left(e_{i},r_{i}\right)\right)$, where $\left(s_{i},D_{i},P_{i},E_{i}\right)$ are feature modalities, $m_{i}$ is the feature mask, and $\left(e_{i},r_{i}\right)$ the labels of enrollment value $e_{i}$ and racial distribution $r_{i}$.

#### Clinical trial site selection

For an input trial, the task is to select *K* sites from its M choices to maximize overall diversity and enrollment. Mathematically, given a trial t and its *M* prospective sites $\left[S_{1},S_{2},\cdots ,S_{M}\right]$, the task becomes to select *K* of those sites based on their present input features to maximize the received reward R(R). In this paper, this is achieved by ranking the *M* sites as R, where $R_{j}$ is the *j*-th site in the ranking, and selecting the *K* highest ranked sites. The ranking R then produces two additional outputs:

(1) $\tilde{e}\in R^{M}$, a vector of the enrollment values ($e_{i}$’s) of each site in the ranked order.
(2) $\tilde{R}\in R^{M\times 6}$, which is the 6 racial distribution values ($r_{i}$’s) for each of the sites in the ranked order.

The notations are summarized in Table 1 for reference.

**Table 1:** Table of notations

Notation	Description
M∈N	The number of site options for a trial
K∈N	The number of sites to select from the *M*
$\lambda \in R^{+}$	The relative weighting of utility and fairness
$t\in R^{n_{t}}$	A vector representation of a clinical trial
$S_{i}$	The *i*-th site option (of *M*) for the trial
$s_{i}\in R^{n_{s}}$	The *i*-th site’s static features modality
$D_{i}\in R^{n_{c}\times n_{d}}$	The *i*-th site’s diagnosis history modality
$P_{i}\in R^{n_{c}\times n_{p}}$	The *i*-th site’s prescription history modality
$E_{i}\in R^{n_{h}\times \left(n_{t}^{\prime }+1\right)}$	The *i*-th site’s enrollment history modality
$m_{i}\in {\left\{0,1\right\}}^{4}$	The *i*-th site’s modality presences
$e_{i}\in N$	The *i*-th site’s enrollment for the trial
$r_{i}\in {\left[0,1\right]}^{6}$	The *i*-th site’s racial distribution vector
*R*	The reward function for a selection of sites
R	An ordered ranking of the *M* sites
$e\in N^{M}$	The site enrollment numbers ordered by R
$R\in {\left[0,1\right]}^{Mx6}$	The racial distributions ordered by R

### Experimental design

We design experiments to evaluate our proposed FRAMM model and answer the following questions:

(1) Is FRAMM effective at enrolling large patient populations in enrollment-only settings?
(2) Can FRAMM make efficient trade-offs between enrollment and diversity to achieve high levels of both?
(3) Does FRAMM improve diversity compared to enrollment-only and post hoc constrained models?

### Experimental setup

#### Datasets

We use IQVIA’s historical real-world clinical trials and claims data in evaluation. The clinical trial database contains 33,323 sites matched with 4,392 trials. We first build the site pool by constructing input features. We then create a separate dataset for each value of *M* that we use. We match each trial to *M* sites, using the top *M* sites (determined by enrollment) in the database if there are enough and otherwise completing the set of *M* by randomly selecting sites from the overall pool and assigning an enrollment of 0 for the trial. Additional details regarding the collection and preprocessing of these data are provided in the supplemental information.

We randomly split these datasets by trial into training and test datasets with an 80-20 ratio so that we may test on true historical data and enrollment counts that are unseen by the compared models. We then further split off 10% of the training set into a validation set. We additionally create a missing data version of the test set in the same way as outlined within our method section by randomly masking each modality with a 20% chance. Using these datasets, we train our models within the PyTorch framework^53^ for 35 epochs at a 0.00001 learning rate and using the Adam optimizer. We save the model that best performs on the validation set as determined by our reward function and evaluate it using the test set.

#### Baselines

We consider the following baseline models.

(1) **Doctor2Vec**^10^ is the current state of the art in enrollment-only trial site selection. It constructs a memory network doctor representation based on static features and patient visits. That representation is then queried by a specific trial representation and fed into a downstream network to predict the doctor’s enrollment count for the trial. Note that Doctor2Vec does not handle missing data and so cannot be trained nor tested on any missing data.
(2) **Random** selects *K* sites at random from the available *M*.
(3) **One-Sides Policy Gradient (PGOS)**^37^ is a fairness baseline that replaces the fairness function *F* with a one-sided loss function that ensures through regularization that groups are not underrepresented within rankings. This baseline represents the typical approach of constraining or regularizing fairness rather than explicitly optimizing diversity. Note that when λ=0, *F* does not contribute to the overall reward, and this is identical to our standard framework. So, it is omitted from any enrollment-only results.

#### Ablation models

To demonstrate FRAMM’s effectiveness at handling missing data and its ability to use missingness as a data augmentation technique to combat low data settings such as ours (the dataset has fewer than 5,000 trials), we add two ablation models without FRAMM’s missing data augmentation and instead trained on the same dataset without any missingness as Doctor2Vec. The first is the full FRAMM model trained on this smaller dataset, and the second removes the missing data mechanism altogether and replaces it with a fully connected layer. We call these models “FRAMM No Missing” and “FC No Missing,” respectively.

#### Metrics

We consider both enrollment and diversity metrics.

For enrollment, we compared the size of each model’s enrolled cohort with the ground truth maximal enrollment via a pair of metrics. First, we use Relative Enrollment Gap calculated by

(Equation 1) $$\text{Relative}\;\text{Enrollment}\;\text{Gap}=\frac{\mathrm{Max}\;\text{Enrollment}-\text{Model}\;\text{Enrollment}}{\mathrm{Max}\;\text{Enrollment}}$$

where max enrollment is the total enrollment from the top K sites after the trial completion (a theoretical ceiling), and model enrollment is the total enrollment from the *K* sites selected by the model. We also report the standard ranking metric normalized Discounted Cumulative Gain (nDCG), defined as

(Equation 2) $$\text{nDCG}=\sum_{j=1}^{K}\frac{2^{m_{j}}-1}{\log_{2}\left(j+1\right)}/\sum_{j=1}^{K}\frac{2^{o_{j}}-1}{\log_{2}\left(j+1\right)}$$

where *m* is the model ranking enrollment list, and *o* is the optimal ranking enrollment list. For example, if we have four sites A, B, C, and D with enrollment values of 5, 10, 8, and 7, respectively, and our model ranked them B → C → A → D, then we would have m=[10,8,5,7] and o=[10,8,7,5].

To measure diversity, we use the entropy of the overall racial distribution of the final enrolled population. This is defined in the same way as above within our reward function, *F*, by

(Equation 3) $$H\left(p\right)=-\sum_{k=1}^{6}p_{k}\;\log\;p_{k}$$

where p is the vector of the proportions of each racial group within the overall enrolled population and so $p_{k}$ is the percentage of a given group.

### Q1. Enrolling large patient populations

We first evaluate each model in enrollment-only settings (with λ=0 for FRAMM variants) to examine their ability to select sites with only enrollment in mind. We display both of our enrollment metrics for the M=20, K=5, λ=0 setting for each compared model in Table 2.

**Table 2:** Enrollment-only performance

	Relative enrollment gap (↓)	nDCG (↑)
Random	0.621 ± 0.019	0.320 ± 0.017
Doctor2Vec	0.525 ± 0.021	0.402 ± 0.018
FRAMM No Missing	0.572 ± 0.020	0.359 ± 0.018
FC No Missing	0.566 ± 0.020	0.363 ± 0.017
FRAMM	0.512 ± 0.020	0.409 ± 0.018

We do see that Doctor2Vec is able to outperform our two ablation, FRAMM-style models trained on the same smaller, full data training dataset of fewer than 5,000 trials without missing data augmentation. However, FRAMM’s missing data mechanism unlocks an effective form of data augmentation that allows it to train on a larger, augmented missing data training dataset. Accordingly, it is able to achieve the best enrollment performance in both metrics on this full data test set, even though it was trained on a different type of data.

### Q2. Making efficient trade-offs

We then showcase our framework’s ability to make effective trade-offs between enrollment and diversity by showing the trajectories of Relative Enrollment Gap vs. Entropy for varying λ values.

**Figure 2:**
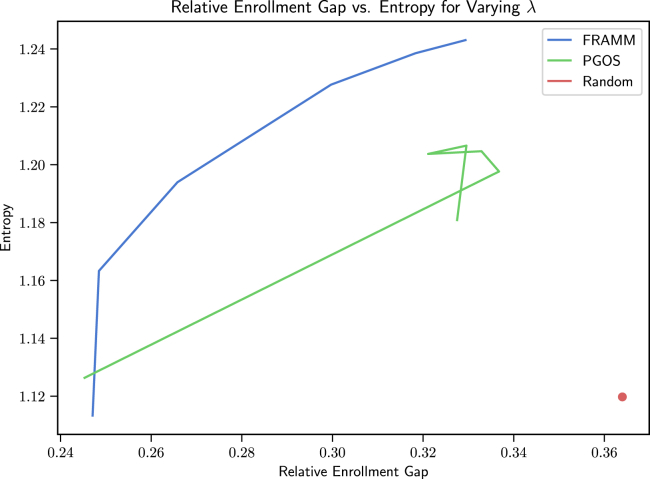
Relative enrollment gap vs. entropy trade-off curves for λ equaling 0.5, 1, 2, 4, and 8 for M=10, K=5 Both FRAMM and the PGOS model are able to increase diversity at the expense of enrollment, but FRAMM makes much more efficient and tunable trade-offs than the PGOS baseline. It maintains much higher enrollment rates for a given level of diversity, achieving a roughly 5% higher peak diversity value and providing up to 9% higher levels of diversity for the same enrollment value.

We compare FRAMM with our PGOS and Random baselines in the M = 10, K = 5 setting on the missing data test set in Figure 2. Here we see that while both FRAMM and the PGOS model greatly outperform the Random baseline and are able to increase diversity at the expense of enrollment, FRAMM makes much more efficient and tunable trade-offs than the PGOS baseline. As such, it maintains much higher enrollment rates for a given level of diversity, achieving a roughly 5% higher peak diversity value and providing up to 9% higher levels of diversity for the same enrollment value. Furthermore, it offers the ability for much more granular tuning through different λ values, whereas the PGOS model is largely constrained to the same region once λ is increased from 0. A version of these results on synthetic data for reproducibility can also be found in Figure S1A in the supplemental information with similar findings.

**Figure 3:**
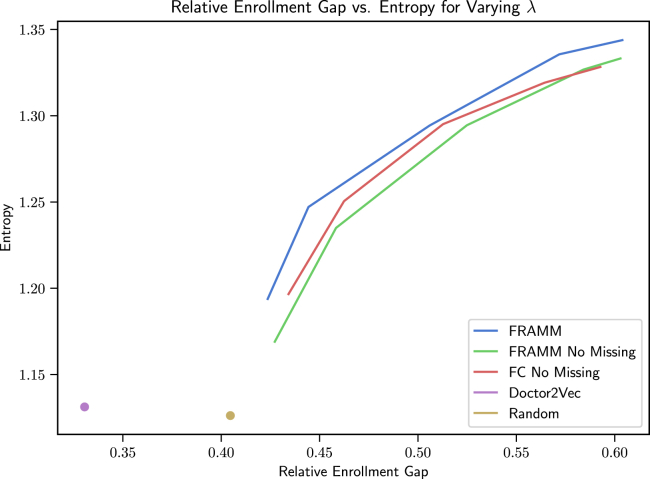
Relative enrollment gap vs. entropy trade-off curves comparing FRAMM with our two ablation models and the two enrollment baselines on the core, no missingness test set FRAMM leverages training with missing data to make more efficient trade-offs than either ablation baseline and achieves more optimal combinations of enrollment and diversity than the Doctor2Vec and Random baselines that are constrained to a single point without any ability to increase diversity.

We also compare FRAMM with our two ablation models and the two enrollment baselines on the core, full data (no missingness) test set in Figure 3. There we see again that FRAMM is effectively leveraging training with missing data in making more efficient trade-offs than either ablation baseline. Finally, we see that it is able to achieve more optimal combinations of enrollment and diversity than the Doctor2Vec and Random baselines, which are constrained to a single point without any ability to increase diversity. A version of these results on synthetic data for reproducibility can also be found in Figure S1B in the supplemental information with similar findings.

### Q3. Improving diversity

Finally, we examine the effect of our model on the enrolled populations of the studies themselves as compared with those selected by the enrollment-only Doctor2Vec model, presenting both the aggregate effect and the effect on a single randomly selected study about multiple sclerosis.

#### Aggregate effect

**Figure 4:**
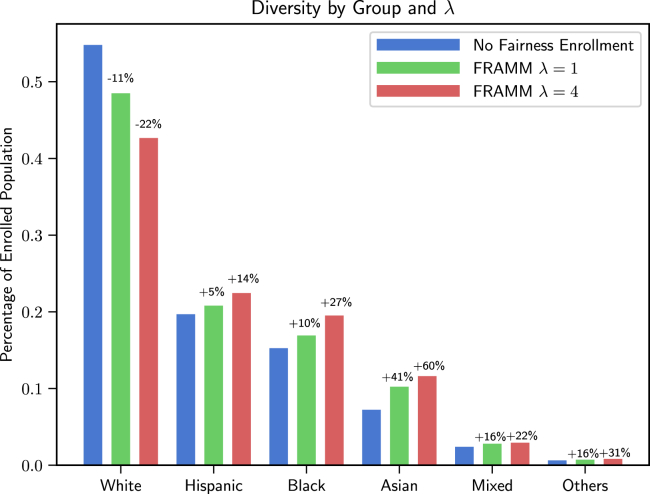
The aggregate effect of different λ values as compared with enrollment of our Doctor2Vec baseline without consideration of fairness on the racial makeup of the average enrolled population We see a dramatic decrease of White enrollment and a clear increase in underrepresented groups.

The aggregate effects are striking in terms of improving diversity. We see a big reduction of the enrollment of the White population with a corresponding increase in each minority group, with the most significant increase for Black and Asian groups. The comparison of the mean percentages of each racial group across the trials in the test set for Doctor2Vec’s chosen cohorts, the population enrolled by FRAMM with λ=1, and the population enrolled by FRAMM with λ=4 can be seen in Figure 4.

#### Individual effect

This effect is mirrored in the case of a single, randomly selected trial for relapsing multiple sclerosis (RMS). We present comprehensive results including all possible site options in Table 3. In comparison with the sites chosen by Doctor2Vec, we see a concerted shift from sites with overwhelmingly White populations to those that enroll just as many but possess much more diversity. Specifically, FRAMM can be seen as extracting the optimal cohort in selecting each of the top five racially diverse sites. As a result, the racial distribution of sites changes from [56.1,15.8,18.1,6.5,2.8,0.7] to [45.9,15.5,26.2,7.0,4.2,0.9], increasing the entropy of the enrolled population from 1.240 to 1.362 while simultaneously enrolling more patients.

**Table 3:** Case study trial site selection

Site location	White	Hispanic	Black	Asian	Mixed	Others	Enrolled
Birmingham, AL	67.7	3.8	25.5	1.7	1.2	0.1	23.0
Wellesley Hills, MA	80.4	4.2	2.0	9.6	3.2	0.5	17.0
Tacoma, WA	60.0	10.9	14.5	5.2	7.8	1.6	16.0
Ann Arbor, MI	66.9	3.7	7.5	17.9	3.9	0.1	16.0
Fort Lauderdale, FL	29.5	25.2	39.4	4.0	1.8	0.1	14.0
San Antonio, TX	53.4	30.8	5.2	8.3	1.6	0.6	12.0
Raleigh, NC	73.3	6.2	10.6	6.5	2.8	0.5	12.0
Kirkland, WA	69.9	8.1	1.4	15.8	4.2	0.7	12.0
Oklahoma City, OK	42.0	3.0	36.1	10.6	6.4	1.9	11.0
Tucson, AZ	77.7	12.4	1.4	5.7	1.8	1.1	11.0
Knoxville, TN	88.2	2.4	2.3	4.1	2.6	0.4	10.0
Charlotte, NC	70.2	2.9	19.5	3.8	2.7	0.9	9.0
Asheville, NC	76.6	9.6	8.0	1.8	3.2	0.9	9.0
Greer, SC	76.1	9.3	7.8	4.9	1.2	0.7	9.0
Cleveland, OH	40.9	2.5	44.7	9.5	2.3	0.2	8.0
Owosso, MI	93.9	3.4	0.5	0.6	1.4	0.3	8.0
Toledo, OH	85.3	3.6	6.0	2.5	1.9	0.6	8.0
Louisville, CO	80.0	6.5	0.4	10.7	2.2	0.2	8.0
Flossmoor, IL	42.5	3.0	50.3	1.3	1.5	1.4	7.0
Cullman, AL	95.2	4.0	0.4	0.2	0.2	0.0	7.0

This performance appears even stronger when put in the context of other options for improving diversity. We already saw in the previous sections that our PGOS fairness baseline made less efficient trade-offs than FRAMM. However, if a trial decided to forgo an RL setup altogether and not optimize for diversity but instead constrain fairness within the selections of an enrollment-only model, the enrollment to achieve that level of diversity would be incredibly low. Specifically in the case of this study, forcing an acceptable level of diversity (for example matching FRAMM’s cohort distribution) on Doctor2Vec’s 5 selected sites through enrollment caps would further reduce its enrollment by at least 18% just to reduce the White population to the same proportion.

## Discussion

In this paper, we proposed a deep RL framework, named FRAMM, for fair trial site selection. Our method uses a missing data mechanism to account for the fact that different modalities of input features can be missing at different sites. It also uses RL with a specifically designed reward function that simultaneously optimizes for both enrollment and fairness to account for the need to make efficient trade-offs between the two objectives. We demonstrate strong performance in achieving state of the-art enrollment levels and also the ability to make efficient and tunable trade-offs between enrollment and diversity. Finally, we show that when diversity is increased, we achieve much more fair site selection in enrolling far more underrepresented populations than other enrollment-only models. While the problem of a lack of diversity in clinical trials is undoubtedly multifaceted and goes far beyond inequitable site selection, we hope our work can bring further attention to this issue, and we believe it is an important step in addressing it head on.

We now discuss a number of limitations regarding our work and our task here. There are a number of realities inherent to our dataset and the site selection task as a whole that may limit the feasibility and effectiveness of our approach.

First, while the dataset we used offers a comprehensive mix of trials across every size and phase as well as the vast majority of medical indications, there may remain some biases based on both the dataset composition and IQVIA’s specific site selection and trial execution process. As such, a model trained on these data should be taken as suited specifically for this dataset and company. Any extrapolation to another setting would require retraining or at least awareness of that fact. However, much like many machine learning tasks and models, we would generally expect FRAMM to be able to learn different patterns for different types of trials at once, despite any compositional disparities, though this should be explored further.

Furthermore, there are some additional nuances on a trial-by-trial basis, such as a small trial running at a limited number of sites, varying eligibility criteria, and geographic constraints, which all can make choosing a more diverse selection more difficult. However, we believe that those challenges require awareness during deployment or use rather than representing an impediment to the feasibility of the task and method in general. Some trials may be unable to successfully use this type of approach (though even geographically or numerically limited sites should still be able to become more diverse even if they struggle to be fully diverse, a reality that makes our approach more attractive than constraint-based methods), but we can still measure the success and ability of a model in the aggregate over a test set of trials as we do here. So, individual trials may have differing levels of both success and ability in terms of population diversification, and we should always be aware of the context when analyzing individual results. However, that reality should not discredit or dishearten our ability to make improvements, even if they are only at the margins or in the aggregate. This work is too important not to.

We strongly believe that it is highly beneficial to create large, publicly available datasets that are de-identified and cross-setting. These datasets can be used to mitigate and analyze the limitations that we have mentioned earlier. Such work can aid in further research and exploration and can help to combat biases. We hope that our work can encourage the creation of such datasets.

## Experimental procedures

### Resource availability

#### Lead contact

Further information and requests for resources should be directed to and will be fulfilled by the lead contact, Jimeng Sun (jimeng@illinois.edu).

#### Materials availability

This study did not generate new unique reagents.

#### Data and code availability

The clinical trial data are publicly available from clinicaltrials.gov, and we share them in processed form. The medical claims (for diagnosis and medication histories) as well as site features and enrollment histories cannot be deposited in a public repository due to privacy and compliance issues. To request access to a processed version of such datasets, please reach out to the lead contact. In addition, we release a synthetic version of the dataset that is generated using the statistical distribution of the real features. All outlined resources along with the code used to run the experiments have been deposited at https://doi.org/10.5281/zenodo.10499129^54^ (representing the repository originally published at https://github.com/btheodorou99/FRAMM) and are publicly available as of the date of publication.

### Fair ranking with missing modalities

Our proposed fair ranking with missing modalities (FRAMM) framework ranks and selects potential trial sites with the following two modules.

(1) **The missing data modality encoders** that generate trial site representations while handling the fact that some of the input site modality embeddings may be missing.
(2) **The scoring and ranking network** that maps each trial site representation to a single score approximating the value of a site to a particular clinical trial before converting the scores into a probability distribution over different rankings according to a learned policy.

### Modality encoding with missing data handling

For each of the *M* sites (where subscript *i* throughout refers to the *i*-th site of the *M*), the modality encoders first embed each available modality and trial representation into a shared representation space, $R^{n_{\text{emb}}}$ (where we use $n_{\text{emb}}=128$).

For the three sequential modalities, diagnosis $D_{i}$, prescription $P_{i}$, and enrollment history $E_{i}$, we embed the inputs using *f*, parameterized for the different settings by *d*, *p*, and *e*, respectively, such that they map $d_{i}^{e}=f_{d}\left(D_{i}\right)$, $p_{i}^{e}=f_{p}\left(P_{i}\right)$, and $e_{i}^{e}=f_{e}\left(E_{i}\right)$. In our experiments, we implement *f* with a bidirectional LSTM followed by a fully connected neural network with ReLU activation function after the first linear layer as in Equation 4.

(Equation 4) f(x)=max(0,biLSTM(x)W+b)V+c

where the LSTM has hidden dimension 128, $W,V\in R^{128\times 128}$, and $b,c\in R^{128}$.

We then embed the static site modality and trial representation using $g_{s}$ and $g_{t}$, respectively, such that they map $s_{i}^{e}=g_{s}\left(s_{i}\right)$ and $t_{i}^{e}=g_{t}\left(t\right)$. We implement *g* as a fully connected neural network with ReLU activation function between layers as in Equation 5,

(Equation 5) g(x)=max(0,xW+b)V+c

where $W\in R^{\dim\left(x\right)\times 128}$, $V\in R^{128\times 128}$, and $b,c\in R^{128}$. So, for each site we have access to four possibly missing modality embeddings $s^{e}$, $d^{e}$, $p^{e}$, and $e^{e}$ and a trial embedding $t^{e}$ all within the shared representation space $R^{n_{\text{emb}}}$. These five embeddings are then fed into the missing data mechanism within the module.

### Missing data handling

To handle missing data modalities, most existing strategies, including modality dropout (MD),^47^ unified representation network (URN),^46^ and cascaded residual autoencoder (CRA)^45^ either do not directly model missing data or require pre-training. To avoid these issues, we use an attention-based approach similar to that in Liu et al.,^49^ which uses the trial embedding as the query for a masked multi-head cross-attention mechanism where the site modality embeddings serve as both keys and values, and the site feature masks dictate whether a given modality can be attended to. The output of this mechanism is the intermediate site embedding, $h_{i}^{\prime }$, which is then concatenated back with the trial embedding to become the trial site representation $h_{i}$. More formally, let the missing data handling procedure be miss such that $h_{i}=miss\left(\left[s_{i}^{e},d_{i}^{e},p_{i}^{e},e_{i}^{e},t_{i}^{e}\right]\right)$. For a given site, this masked cross-attention approach arrives at $h_{i}$ by Equation 6,

(Equation 6) $$\begin{array}{l} h_{i}^{\prime }=\text{att}\left(t_{i}^{e},\left[s_{i}^{e},d_{i}^{e},p_{i}^{e},e_{i}^{e}\right],\left[s_{i}^{e},d_{i}^{e},p_{i}^{e},e_{i}^{e}\right]\right)\\ \text{att}\left(Q,K,V\right)=\text{concat}\left({\text{head}}_{1},\cdots ,{\text{head}}_{4}\right)W^{O}\\ {\text{head}}_{j}=\sigma \left(\frac{\left(QW_{j}^{Q}\right){\left(KW_{j}^{K}\right)}^{T}}{\sqrt{n_{emb}/n_{\text{head}}}}+m_{i}^{\prime }\right)\left(VW_{j}^{V}\right)\\ m_{ik}^{\prime }=\left\{ \begin{array}{cc} 0 & m_{ik}=1\\ -\infty & m_{ik}=0 \end{array}\right.\\ h_{i}=\text{concat}\left(h_{i}^{\prime },t_{i}^{e}\right) \end{array}$$

where σ is the softmax function, $W_{j}^{Q},W_{j}^{K},W_{j}^{V},W^{O}\in R^{128\times 128}$, and $m_{i}^{\prime }\in R^{4}$ is a conversion of the *i*-th site’s binary mask vector into the form required for masking in attention. That conversion converts a mask value of 0 (signifying a missing modality) to −∞ to prevent any remaining attention weight after a softmax function over the sum of the values and masks, and it converts a value of 1 (signifying a present modality) to 0 to not interfere with the softmax calculation.

### Missing data augmentation

This missing data handling also unlocks a new form of data augmentation that we incorporate directly into the FRAMM framework. For any data we aim to train a FRAMM model on, we may augment the dataset by repeating each trial data point while randomly discarding or masking each site modality. Specifically, we create 10 versions of each trial, and for each site in each version of each trial we randomly create a mask that prevents the architecture from using certain modalities. To do this, we set a 20% chance of each modality being missing, and we stipulate that if a modality is truly missing it must be masked but also necessitate that at least one modality must remain unmasked. In this way, FRAMM’s missing data handling allows it to not only adapt to different settings and varying input features but also augment any training data to achieve better performance regardless of the underlying missingness.

### Deep Q-network and ranking policy

The second module takes the *M* trial site representations, now without any missingness, and uses them to rank the sites. To do so, it first maps the representations $H=\left[h_{1}\cdots h_{M}\right]$ to *M* real-valued scores $q\in R^{M}$, which approximate the value each site will provide toward the final reward obtained by selecting them for the current trial. The q here serves an analogous (although not identical) role to a Q-Value in RL as in Equation 7,

(Equation 7) $$Q\left(s,a\right)=R\left(s,a\right)+\gamma \max_{a^{\prime }}Q\left(s^{\prime },a^{\prime }\right)$$

This mapping is denoted generally by the score function in Equation 8,

(Equation 8) q=score(H)

### Reward function

The reward function used in this paper and which the Q-Network learns to approximate consists of two components for enrollment utility and fairness objectives and is defined by Equation 9,

(Equation 9) R(R)=U(R)+λF(R)

where λ is a hyperparameter determining the relative weighting of the two reward components. This relative weighting forms a trade-off such that multiple λ values can be utilized and the desired point along the trade-off curve can be selected. We chose λ as 0, 0.5, 1, 2, 4, or 8 in our experiments.

The utility component is defined by Equation 10,

(Equation 10) $$U\left(R\right)=\frac{\sum_{j=1}^{K}{\tilde{e}}_{j}-\sum_{j=K+1}^{M}{\tilde{e}}_{j}}{\sum_{j=1}^{M}{\tilde{e}}_{j}}$$

which is the enrollment difference between the chosen sites (top-*K* sites) and those not (the remaining M−K sites), normalized for the total enrollment numbers.

The fairness component is the entropy of the racial distribution of the total patient population enrolled by the *K* chosen sites, formally defined by Equation 11,

(Equation 11) $$F\left(R\right)=H\left(\frac{\sum_{j=1}^{K}{\tilde{e}}_{j}{\tilde{r}}_{j}}{\sum_{j=1}^{K}{\tilde{e}}_{j}}\right)$$

where both ${\tilde{e}}_{j}$ (the enrollment values in the ranked order) and the bottom sum are scalars applied to the entire 6-dimensional vector ${\tilde{r}}_{j}$, the top sum operates element-wise over those vectors, and *H* is the standard definition of entropy where for a probability distribution n (such as the one outputted by our sum), $H\left(n\right)=-\sum_{k}n_{k}\;\log\;n_{k}$.

### Q-network architecture

We implement this mapping using a transformer encoder layer with a fully connected head. We postulate that transformer layers and their ability for each site’s score to be affected by the other sites available can be especially valuable given the dynamic nature of *F* in which the fairness component of our reward evaluates all of the selected sites together rather than acting as a sum of functions of each individual site as with the utility component. The generation of the scores is formally given by Equation 12,

(Equation 12) $$\begin{array}{l} H^{\left(0\right)}=H\\ H^{\prime \left(i\right)}=\mathrm{LN}\left(H^{\left(i-1\right)}+\text{att}\left(H^{\left(i-1\right)},H^{\left(i-1\right)},H^{\left(i-1\right)}\right)\right)\\ H^{\left(i\right)}=\mathrm{LN}\left(H^{\prime \left(i\right)}+\left(\max\left(0,H^{\prime \left(i\right)}W^{\left(i\right)}+b^{\left(i\right)}\right)V^{\left(i\right)}+c^{\left(i\right)}\right)\right)\\ q=\max\left(0,H^{\left(n_{l}\right)}W^{f}+b^{f}\right)V^{f}+c^{f} \end{array}$$

where $i\in \left\{1,\cdots ,n_{l}\right\}$, LN denotes layer normalization, $W^{\left(i\right)},V^{\left(i\right)}\in R^{128\times 128}$, $b^{\left(i\right)},c^{\left(i\right)}\in R^{128}$, $W^{f}\in R^{128\times 64}$, $b^{f}\in R^{64}$, $V^{f}\in R^{64\times 1}$, and $c^{f}\in R$.

### Ranking and policy learning

These “Q-Value” scores are then fed into the final portion of our framework where they are used to generate rankings and their probabilities using a non-deterministic policy π. While simply selecting the top *K* scores represents the best site selection as currently approximated by the network (and is used for testing), it prevents exploration of the ranking space during training. Instead, we define our stochastic policy by Equation 13,

(Equation 13) $$\pi \left(R\right)=\sum_{R^{\prime }\in \varphi \left(R\right)}\prod_{j=1}^{K}\frac{\;\exp\;\left(q\left(R_{j}^{\prime }\right)\right)}{\sum_{k=j}^{M}\;\exp\;\left(q\left(R_{k}^{\prime }\right)\right)}$$

where φ maps to the set of other rankings that are the same as R except for permuting the first *K* elements, and the *q* function returns the score given by the Q-Value network for the given site. Our policy’s probability represents the odds of a given top-*K* combination (where order does not matter) achieved by sampling *K* sites without replacement according to the softmax probabilities defined by their scores. However, in practice, this quickly becomes impractical for any sizable *K* as the permutation space grows factorially. Instead, we use an unbiased estimate of the probability of a top-*K* combination calculated for a given ranking by randomly permuting the order of the first *K* elements (to remove the bias of higher probability rankings in the permutation space being drawn more often), calculating the product in the policy’s equation, and scaling by K!. In this way, we are able to estimate according to the expectation in Equation 14,

(Equation 14) $$\pi \left(R\right)=\sum_{R^{\prime }\in \varphi \left(R\right)}\prod_{j=1}^{K}\frac{\;\exp\;\left(q\left(R_{j}^{\prime }\right)\right)}{\sum_{k=j}^{M}\;\exp\;\left(q\left(R_{k}^{\prime }\right)\right)}=K!\underset{R^{\prime }\in \varphi \left(R\right)}{E}\prod_{j=1}^{K}\frac{\;\exp\;\left(q\left(R_{j}^{\prime }\right)\right)}{\sum_{k=j}^{M}\;\exp\;\left(q\left(R_{k}^{\prime }\right)\right)}$$

Given our architecture for obtaining scores for each site, and our method of sampling and obtaining probability estimates of rankings given those scores, all that is left is to handle optimization and policy learning. The overall goal is to maximize the expected reward in Equation 15,

(Equation 15) $$E_{R∼\pi }\left[R\left(R\right)\right]$$

We implement this using REINFORCE.^55^ It is a common policy gradient algorithm that directly optimizes the expected reward as calculated through Monte Carlo sampling weighted by the log-likelihoods of rankings. Using this or any other method policy gradient algorithm, we are able to use backpropagation back through our policy, Q-Network, and modality encoders to train our framework via real-world enrollment and diversity data.

### Assumptions regarding randomness

Finally, we conclude our presentation of FRAMM by discussing some minutiae regarding the assumptions and flexibility of FRAMM’s augmentation approach as well as providing some brief experimental results demonstrating its value even if those assumptions are not upheld.

### Different patterns of randomness

FRAMM’s augmentation is currently constructed to follow the pattern of modalities missing completely at random (MCAR). However, there are many different patterns of missingness that may exist in real-world sites, and this augmentation design could be adjusted if the pattern of missingness is known or at least suspected. Furthermore, if true missing modality data are available, FRAMM may be trained on that as well. Regardless, even if the true pattern of missingness is unknown, and we use this MCAR approach, which does not match the true pattern, we believe the augmentation helps.

### Handling serially missing modalities

**Figure 5:**
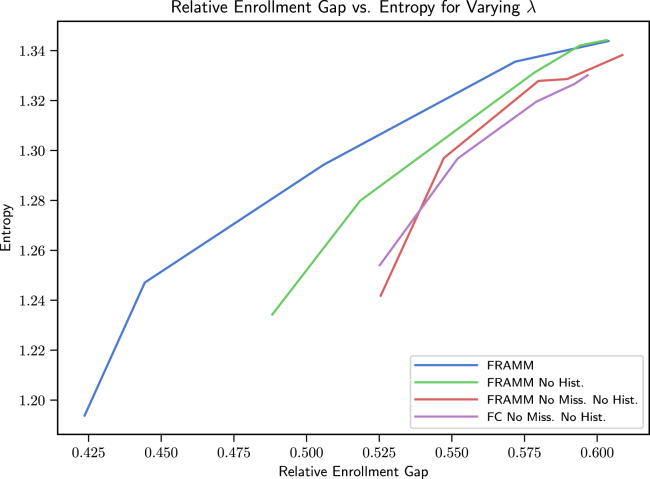
Visualizations of relative enrollment gap vs. entropy trade-off curves for λ equaling 0.5, 1, 2, 4, and 8 for the missing modality experiment (with the core FRAMM model evaluated on the full data test set as a gold standard benchmark) for M=20, K=5 While each of the three compared models suffers a drop-off compared with the full data results, the FRAMM framework trained on a larger missing data dataset outperforms the other two by a sizable margin and approaches the full data performance for larger λ.

To that end, we show the ability of our framework to be robust in adapting to new settings with different data availability such as would be found in a new healthcare system with access to different types of data. Specifically, we explore the performance of FRAMM in the face of an unexpected serially missing modality, which appears in training but then is completely missing during testing. We do this by testing FRAMM and our two ablation baselines not on the full data test set but instead the same test set with the enrollment history modality missing for each investigator in each trial. We show the trade-off results of this experiment in the M=20, K=5 setting with varying λ in Figure 5. While each suffers a drop-off in comparison with the best evaluation on the full data test set, we see that our model clearly outperforms both of the models that were trained solely on the full data, reducing the enrollment gap from that full data benchmark nearly in half for similar levels of diversity and approaching the full data performance for large λ.

## Supplemental information

Document S1. Figure S1, Table S1, and supplemental experimental procedures

Document S2. Article plus supplemental information

## References

[1] Sharma A., Palaniappan L. (2021). Improving diversity in medical research. Nat. Rev. Dis. Prim..

[2] Hughson J.-a., Woodward-Kron R., Parker A., Hajek J., Bresin A., Knoch U., Phan T., Story D. (2016). A review of approaches to improve participation of culturally and linguistically diverse populations in clinical trials. Trials.

[3] Knepper T.C., McLeod H.L. (2018). When will clinical trials finally reflect diversity?. Nature.

[4] Nephew L.D. (2021). Accountability in clinical trial diversity: The buck stops where?. EClinicalMedicine.

[5] Yates I., Byrne J., Donahue S., McCarty L., Mathews A. (2020). Representation in clinical trials: A review on reaching underrepresented populations in research. Clin. Res..

[6] Burroughs V.J., Maxey R.W., Levy R.A. (2002). Racial and ethnic differences in response to medicines: towards individualized pharmaceutical treatment. J. Natl. Med. Assoc..

[7] Hwang T.J., Brawley O.W. (2022). New federal incentives for diversity in clinical trials. N. Engl. J. Med..

[8] Liu R., Rizzo S., Whipple S., Pal N., Pineda A.L., Lu M., Arnieri B., Lu Y., Capra W., Copping R., Zou J. (2021). Evaluating eligibility criteria of oncology trials using real-world data and ai. Nature.

[9] Gray D.M., Nolan T.S., Gregory J., Joseph J.J. (2021). Diversity in clinical trials: an opportunity and imperative for community engagement. Lancet. Gastroenterol. Hepatol..

[10] Biswal S., Xiao C., Glass L.M., Milkovits E., Sun J. (2020).

[11] (2020). Mdgroup. The True Cost of Patient Drop-Outs in Clinical Trials. https://mdgroup.com/blog/the-true-cost-of-patient-drop-outs-in-clinical-trials/.

[12] Gao J., Xiao C., Glass L.M., Sun J.C. (2020). Proceedings of the 26th ACM SIGKDD International Conference on Knowledge Discovery & Data Mining.

[13] Zhang X., Xiao C., Glass L.M., Sun J.D. (2020). Patient-trial matching with deep embedding and entailment prediction. Proceedings of The Web Conference.

[14] Theodorou B.P., Xiao C., Sun J.T. (2023). Proceedings of the 14th ACM International Conference on Bioinformatics, Computational Biology, and Health Informatics.

[15] Wang Z., Sun J. (2022).

[16] Fu T., Huang K., Xiao C., Glass L.M., Sun J. (2022). Hint: Hierarchical interaction network for clinical-trial-outcome predictions. Patterns.

[17] Gligorijevic J., Gligorijevic D., Pavlovski M., Milkovits E., Glass L., Grier K., Vankireddy P., Obradovic Z. (2019). Optimizing clinical trials recruitment via deep learning. J. Am. Med. Inf. Assoc..

[18] Chouldechova A., Roth A. (2018). The frontiers of fairness in machine learning. arXiv.

[19] Mehrabi N., Morstatter F., Saxena N., Lerman K., Galstyan A. (2021). A survey on bias and fairness in machine learning. ACM Comput. Surv..

[20] Caton S., Haas C. (2020). Fairness in machine learning: A survey. arXiv.

[21] Zhang B.H., Lemoine B., Mitchell M. (2018). Proceedings of the 2018 AAAI/ACM Conference on AI, Ethics, and Society.

[22] Joseph M., Kearns M., Morgenstern J., Roth A. (2016). Fairness in learning: Classic and contextual bandits. arXiv.

[23] Feldman M., Friedler S.A., Moeller J., Scheidegger C., Venkatasubramanian S. (2015). Proceedings of the 21st ACM SIGKDD International Conference on Knowledge Discovery & Data Mining.

[24] Jiang H., Nachum O. (2020). International Conference on Artificial Intelligence and Statistics.

[25] Zemel R., Wu Y., Swersky K., Pitassi T., Dwork C. (2013). International conference on machine learning.

[26] Nabi R., Malinsky D., Shpitser I. (2019). International Conference on Machine Learning.

[27] Celis L.E., Huang L., Keswani V., Vishnoi N.K. (2019). Proceedings of the conference on fairness, accountability, and transparency.

[28] Narasimhan H. (2018). International Conference on Artificial Intelligence and Statistics.

[29] Xia F., Liu T.-Y., Li H. (2009). Advances in Neural Information Processing Systems.

[30] Richardson M., Prakash A., Brill E. (2006). Proceedings of the 15th international conference on World Wide Web.

[31] Rahangdale A., Raut S. (2019). Machine learning methods for ranking. Int. J. Software Eng. Knowl. Eng..

[32] Zehlike M., Sühr T., Baeza-Yates R., Bonchi F., Castillo C., Hajian S. (2022). Fair top-k ranking with multiple protected groups. Inf. Process. Manag..

[33] Asudeh A., Jagadish H., Stoyanovich J., Das G. (2019). Proceedings of the 2019 International Conference on Management of Data.

[34] Yadav H., Du Z., Joachims T. (2021). Proceedings of the 44th International ACM SIGIR Conference on Research and Development in Information Retrieval.

[35] Ghosh A., Dutt R., Wilson C. (2021). Proceedings of the 44th international ACM SIGIR conference on research and development in information retrieval.

[36] Ge Y., Liu S., Gao R., Xian Y., Li Y., Zhao X., Pei C., Sun F., Ge J., Ou W. (2021). Proceedings of the 14th ACM International Conference on Web Search and Data Mining.

[37] Singh A., Joachims T. (2019). Policy learning for fairness in ranking. Adv. Neural Inf. Process. Syst..

[38] Wei Z., Xu J., Lan Y., Guo J., Cheng X. (2017). Proceedings of the 40th International ACM SIGIR Conference on Research and Development in Information Retrieval.

[39] Zhou J., Agichtein E.R. (2020). Proceedings of The Web Conference.

[40] Liu W., Liu F., Tang R., Liao B., Chen G., Heng P.A. (2020). Balancing between accuracy and fairness for interactive recommendation with reinforcement learning. Advances in Knowledge Discovery and Data Mining.

[41] Feng J., Li H., Huang M., Liu S., Ou W., Wang Z., Zhu X. (2018). Proceedings of the 2018 World Wide Web Conference.

[42] Clark K., Manning C.D. (2016). Proceedings of the 2016 Conference on Empirical Methods in Natural Language Processing.

[43] Wei J., Zeng A., Wu Y., Guo P., Hua Q., Cai Q. (2020). Generator and critic: A deep reinforcement learning approach for slate re-ranking in e-commerce. Arxiv.

[44] Ma M., Ren J., Zhao L., Tulyakov S., Wu C., Peng X.S. (2021). Proceedings of the AAAI Conference on Artificial Intelligence.

[45] Tran L., Liu X., Zhou J., Jin R. (2017). Proceedings of the IEEE Conference on Computer Vision and Pattern Recognition.

[46] Lau K., Adler J., Sjölund J. (2019). A unified representation network for segmentation with missing modalities. arXiv.

[47] Parthasarathy S., Sundaram S. (2020). Companion Publication of the 2020 International Conference on Multimodal Interaction.

[48] Ma F., Xu X., Huang S.-L., Zhang L. (2021). Maximum likelihood estimation for multimodal learning with missing modality. arXiv.

[49] Liu L., Liu S., Zhang L., To X.V., Nasrallah F., Chandra S.S. (2023). Cascaded multi-modal mixing transformers for alzheimer’s disease classification with incomplete data. Neuroimage.

[50] Ma M., Ren J., Zhao L., Testuggine D., Peng X. (2022). Proceedings of the IEEE/CVF Conference on Computer Vision and Pattern Recognition.

[51] Qian S., Wang C. (2023). Com: Contrastive masked-attention model for incomplete multimodal learning. Neural Network..

[52] (2018). https://www.cdc.gov/antibiotic-use/community/pdfs/Uniform-System-of-Classification-2018-p.pdf.

[53] Paszke A., Gross S., Massa F., Lerer A., Bradbury J., Chanan G., Killeen T., Lin Z., Gimelshein N., Antiga L. (2019). http://papers.neurips.cc/paper/9015-pytorch-an-imperative-style-high-performance-deep-learning-library.pdf.

[54] (2024).

[55] Williams R.J. (1992). Simple statistical gradient-following algorithms for connectionist reinforcement learning. Mach. Learn..

