# Supplementary material for: FRAMM: Fair ranking with missing modalities for clinical trial site selection
Source: Patterns (N Y). 2024 Mar 1;5(3):100944. doi: 10.1016/j.patter.2024.100944 (PMC10935501; doi:10.1016/j.patter.2024.100944)
Supplement: Document S1. Figure S1, Table S1, and supplemental experimental procedures [file mmc1.pdf]

Patterns, Volume *5*

Supplemental information

FRAMM: Fair ranking with missing modalities for clinical trial site selection

Brandon Theodorou, Lucas Glass, Cao Xiao, and Jimeng Sun

We aim for our main paper to be self-contained and comprehensive regarding our task, our proposed FRAMM framework, and its results. However, we add and discuss a number of ideas here to ensure that we provide a full set of information regarding our work and especially regarding its reproducibility. To that end, here we provide additional experimental details for the results in our main paper and also outline the creation of a synthetic version of our dataset that we have released at https://github.com/btheodorou99/FRAMM[1].

Supplemental Experimental Procedures

We first discuss some concrete details about the data preparation used to create our real-world dataset and then the setup and hyperparameters that went into model training and validation.

Input Feature Specifics

We outline the features that compose the representation vectors and matrices for the trial and site representations in our main paper, but we provide additional details regarding their construction and dimensionality here. n_t and n_s, representing the dimensionality of the trial feature vector and the static site feature vector respectively, are equal to 1827 and 669. The one-hot vectors representing a site's diagnosis history have dimensionality $n_d = 260$ for ICD-10 code categories defined by the first letter and second number of a given code, representing its higher-level category in the wider ICD-10 ontology. Similarly, the prescription history one-hot vectors have dimensionality $n_r = 100$ for the first two digits in each code within the USC ontology[2].

Data Source and Preparation

For our experiments, we use real-world clinical trials and claims data to train and evaluate our algorithm. We collect this data from IQVIA's internal historical records regarding trials that they have run within the United States and sites that they have contracted with. The clinical trial database contains 33,323 sites matched with 4,392 trials.

To process this data, we first build the site pool for each site, creating the static features, matching the 500 most recent diagnoses and prescriptions, and setting the enrollment histories for the 50 most recent trials at any given point in time. We then create a separate dataset for each value of M that we use. We match each trial to M sites, using the top M sites (determined by enrollment) in the database if there are enough and otherwise completing the set of M by randomly selecting sites from the overall pool and assigning an enrollment of 0 for the trial. Finally, for the FRAMM model, we perform missing data augmentation by creating 10 versions of each trial, and for each site in each trial randomly creating a mask that dictates whether a given site modality is present for that data point, where each modality has an 80% chance of being present, and we stipulate that at least one modality must be present for each site.

Model training and validation

We then split this dataset into training, validation, and test datasets. Each split is random, with 20% of the overall dataset being reserved for use as a test set, and 10% of the remaining training set serving as our validation set. We also create a missing data version of the test set in the same way as we performed missing data augmentation for FRAMM's training set. We train our models within the PyTorch framework[3] for 35 epochs at a 0.00001 learning rate and using the Adam

Table S1: Synthetic Enrollment-Only Performance

	Relative Enrollment Gap	nDCG
Random	0.227 ± 0.003	0.707 ± 0.003
FRAMM	0.062 ± 0.001	0.922 ± 0.002

optimizer. We save the model that best performs on the validation set as determined by our loss function and evaluate it using the two test sets.

Synthetic Dataset

We now outline the creation of and results on the synthetic dataset which was built to offer reproducibility of our work. All of our code for these experiments can be found at `https://github.com/btheodorou99/FRAMM`[1].

Dataset Creation

The trial data itself is not proprietary as it was scraped from the publicly available `clinicaltrials.gov` website. So, we begin with the true trial representations. We then build a pool of 30,000 sites as follows. We first randomly build each of the static modality features except for the primary specialty and racial distribution. The primary specialty is sampled from the percentages of each specialty within the true dataset, and the racial demographic distribution values are sampled from normal distributions centered roughly at the aggregate distributions from the true dataset and normalized. All of the features are then concatenated to form the static features modality for each synthetic site. We then build the diagnosis and prescription history modalities by sampling bigram probabilities for each of the 500 values from the true dataset values (with the initial value conditioning the first bigram being the primary specialty).

Now that we have our trials and our pool of sites (they each start with no enrollment history), we move on to simulating trials in order to generate the enrollment history and full synthetic dataset. We first train a model on the real dataset to predict the enrollment of a site for a given trial. This model acts as our data labeler. We then randomly order the trial representations before proceeding through them sequentially to generate the data. For each trial, we randomly select 20 sites from the pool and pass them in (with their current enrollment history) to the labeler. The labeler's outputs are used as the enrollment labels and combined with the site modalities and trial representation to build a single data point. We update each site's enrollment history with the trial representation and enrollment label and continue to the next trial. The code to build this dataset (and necessary artifacts such as the labeler model and bigram probabilities) are all available within the GitHub repository we provide at `https://github.com/btheodorou99/FRAMM`.

Synthetic Results

We now present our results on the synthetic dataset. We train each of our compared models on the $M = 20$, $K = 10$ setting for each of our λ values (0, 0.5, 1, 2, 4, and 8) in order to generate a full set of results that qualitatively mirror those from the real-world dataset and are outlined below.

Enrolling Large Patient Populations The enrollment-only results for $\lambda = 0$ on the synthetic dataset once again demonstrate a huge level of improvement over the random enrollment base-

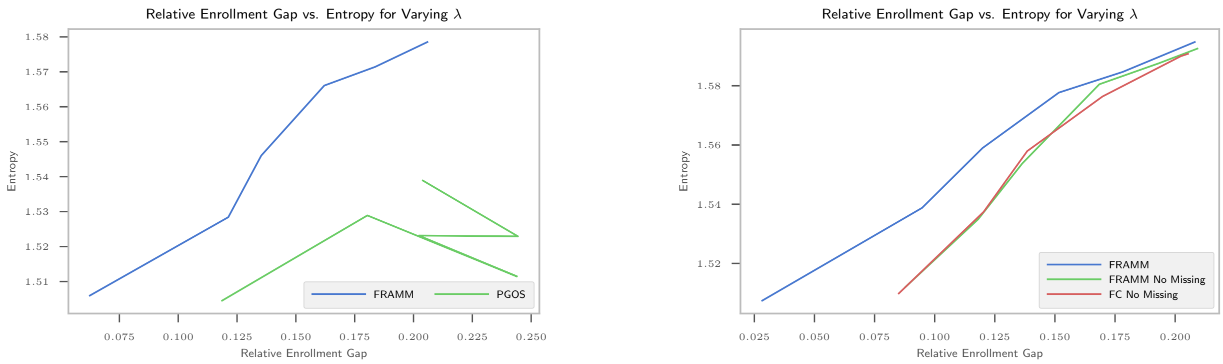

(a) Missing Data Test Set

(b) Full Data Test Set

Figure S1: Visualizations of Relative Enrollment Gap vs. Entropy trade-offs for $M = 20$, $K = 10$, and λ equaling 0, 0.5, 1, 2, 4, and 8 in each of our test settings on the synthetic dataset. Figure (a) compares `FRAMM` and the PGOS baseline on the core missing data test set where `FRAMM` is able to make much more efficient and tunable trade-offs than the PGOS baseline. Figure (b) shows the data augmentation experiment. While it was not trained on the complete (non-missing) data each model was evaluated on, we see a clear improvement via the `FRAMM` framework trained on a larger missing data dataset.

line. `FRAMM` here reduces Relative Enrollment Gap up to 74% on the missing data test set. The full results of this experiment can be seen in Table S1.

Balancing Enrollment and Fairness Trade-off The ability of different models to make effective trade-offs between enrollment and diversity on the synthetic dataset via varying λ values is then shown in Figure S1a. `FRAMM` is generally able to make much more efficient and tunable trade-offs between our two objectives than the PGOS baseline. Furthermore, we show the boost from `FRAMM`'s missing data augmentation on the synthetic dataset. We display the trade-off results in Figure S1b where we see that `FRAMM` easily outperforms the two ablation variants trained only on the smaller, complete dataset. We specifically note that the `FRAMM` model trained on missing data is especially effective in more enrollment-based settings, outperforming the baselines for all λ but reducing Relative Enrollment Gap up to 84% when $\lambda = 0$.

Supplemental References

1. btheodorou99. btheodorou99/framm: First release (2024). URL: `https://doi.org/10.5281/zenodo.10499129`. doi:.

2. The uniform system of classification (usc). Report Centers for Disease Control and Prevention (2018). URL: `https://www.cdc.gov/antibiotic-use/community/pdfs/Uniform-System-of-Classification-2018-p.pdf`.

3. Paszke, A., Gross, S., Massa, F., Lerer, A., Bradbury, J., Chanan, G., Killeen, T., Lin, Z., Gimelshein, N., Antiga, L., Desmaison, A., Kopf, A., Yang, E., DeVito, Z., Raison, M., Tejani, A., Chilamkurthy, S., Steiner, B., Fang, L., Bai, J., and Chintala, S. Pytorch: An imperative style, high-performance deep learning library. In: *Advances in Neural Information Processing Systems 32* (8024–8035). Curran Associates, Inc. (2019):(8024–8035). URL: `http://papers.neurips.cc/paper/9015-pytorch-an-imperative-style-high-performance-deep-learning-library.pdf`.
